# Intracellular Calcium Disturbances Induced by Arsenic and Its Methylated Derivatives in Relation to Genomic Damage and Apoptosis Induction

**DOI:** 10.1289/ehp.7634

**Published:** 2005-02-10

**Authors:** Ana-Maria Florea, Ebenezer N. Yamoah, Elke Dopp

**Affiliations:** ^1^Institute of Hygiene and Occupational Medicine, University Hospital, Essen, Germany; ^2^Department of Otolaryngology, Center for Neuroscience, University of California, Davis, California, USA

**Keywords:** apoptosis, arsenic, genomic damage, intracellular calcium

## Abstract

Arsenic and its methylated derivatives are contaminants of air, water, and food and are known as toxicants and carcinogens. Arsenic compounds are also being used as cancer chemotherapeutic agents. In humans, inorganic arsenic is metabolically methylated to mono-, di-, and trimethylated forms. Recent findings suggest that the methylation reactions represent a toxification rather than a detoxification pathway. In recent years, the correlation between arsenic exposure, cytotoxicity and genotoxicity, mutagenicity, and tumor promotion has been established, as well as the association of arsenic exposure with perturbation of physiologic processes, generation of reactive oxygen species, DNA damage, and apoptosis induction. Trivalent forms of arsenic have been found to induce apoptosis in several cellular systems with involvement of membrane-bound cell death receptors, activation of caspases, release of calcium stores, and changes of the intracellular glutathione level. It is well known that calcium ion deregulation plays a critical role in apoptotic cell death. A calcium increase in the nuclei might lead to toxic effects in the cell. In this review, we highlight the relationship between induced disturbances of calcium homeostasis, genomic damage, and apoptotic cell death caused by arsenic and its organic derivatives.

## Arsenic and Its Derivatives as Potent Environmental Toxicants

Exposure to high levels of arsenic in drinking water has been recognized for many decades in some regions of the world, notably in China, India, and some countries in Central and South America. Millions of people are at risk of cancer and other diseases because of chronic arsenic exposure (National Research Council 1999, 2001).

General adverse health effects that are associated with human exposure to arsenicals include cardiovascular diseases, developmental abnormalities, neurologic and neurobehavioral disorders, diabetes, hearing loss, fibrosis of the liver and lung, hematologic disorders, blackfoot disease, and cancers (Abernathy et al. 1999; Sordo et al. 2001; Tchounwou et al. 1999). In humans, arsenic is known to cause cancer of the skin (in combination with ultraviolet irradiation; Rossman et al. 2004) and cancer of the lung, bladder, liver, and kidney (Abernathy et al. 1999; Kitchin 2001; Tchounwou et al. 1999). The principal proposed mechanisms of arsenic carcinogenicity are induction of chromosomal abnormalities, promotion, and oxidative stress (Kitchin 2001; Kitchin and Ahmad 2003). Also, chronic exposure to arsenic has been found to cause immunotoxicity and has been associated with the suppression of hematopoiesis (anemia and leukopenia; Cheng et al. 2004). In its inorganic form, arsenic is known to be cytotoxic and genotoxic *in vivo* and *in vitro* (for review, see Dopp et al. 2004a).

**Figure 1:**
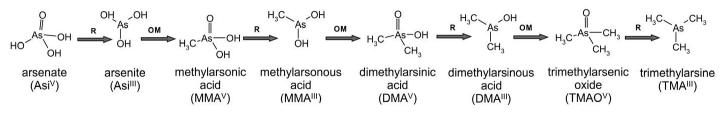
Challenger (1945) mechanism for arsenic biomethylation. R = reduction, OM = oxidative methylation. [Copyright 2004 from “Environmental Distribution, Analysis and Toxicity of Organometal(loid) Compounds” by Dopp et al. (2004a). Reproduced by permission of Taylor & Francis Group, LLC. (http://www.taylorandfrancis.com).]

Inorganic arsenic is methylated via glutathione (GSH) conjugation to the pentavalent species: monomethylarsonic acid [MMA(V)], dimethylarsinic acid [DMA(V)], and tri-methylarsenic oxide [TMAO(V)] (Kitchin 2001; Sordo et al. 2001). This process requires the metabolic reduction of As(5^+^) to As(3^+^), and in this way, trivalent monomethylarsonous acid [MMA(III)], dimethylarsinous acid [DMA(III)], and trimethylarsine [TMA(III)] appear as metabolic products (Kitchin 2001; Kitchin and Ahmad 2003; Sordo et al. 2001) (Figure 1). Recent findings show that the trivalent methylated arsenic metabolites are highly toxic; DMA(III) has been shown to cause several genotoxic and/or clastogenic effects such as single-strand breaks, formation of apurinic/apyrimidinic sites, DNA and oxidative base damages, DNA–protein cross-links, chromosomal aberrations, and aneuploidy (Dopp et al. 2004b; Schwerdtle et al. 2003; Sordo et al. 2001). The genotoxic effects of arsenic and its methylated metabolites *in vivo* and *in vitro*, as well as the carcinogenic potencies of these substances, are discussed in detail by Dopp et al. (2004a), Florea et al. (2004), Patrick (2003), Hughes (2002), and Gebel (2001).

The major mechanisms in which toxic metallic entities may damage cells are direct binding to cellular molecules, induction of conformational changes, replacement of physiologic metals from their binding sites (Qian et al. 2003), or inhibition of DNA repair functions (Hartwig 1998). Thus, they may act as catalysts for the redox reactions that produce reactive oxygen species (ROSs). ROSs are capable of damaging a wide variety of cellular macromolecules, including DNA, lipids, and proteins. Finally, cellular signal transduction can be altered (e.g., activation of transcription factors, changes of gene expression); cell growth, proliferation, and differentiation can be promoted; and apoptosis leading to cell death or cancer development can be induced (Qian et al. 2003; Yang and Frenkel 2002).

In addition, Murphy et al. (1981) suggested a neurotoxic potential of arsenic after acute arsenic intoxication of human patients that caused a polyneuropathy with prolonged sensory and motor deficits. Namgung and Xia (2001) have shown that primary cultures of rat cerebellar neurons exposed to 5–15 μM sodium arsenite and 1–5 mM DMA(V) had reduced viability. These authors reported nuclear fragmentation, DNA degradation, and apoptosis induction in neuronal cells treated with sodium arsenite or DMA(V). They concluded that the neurotoxicity of arsenite might be caused by an activation of p38 and c-*Jun* N-terminal kinase 3 (JNK3) mitogen-activated protein kinases (MAPKs), which are involved in the apoptotic process.

The role of metallothionein (MT) in modifying DMA(V) genotoxicity was recently studied in MT-I/II null mice and in the corresponding wild-type mice by Jia et al. (2004). In this study, increased formation of 8-hydroxy-2′-deoxyguanosine was found together with elevated numbers of DNA strand breaks. The observed levels were significantly higher in MT-I/II null mice than in wild-type mice. Furthermore, the appearance of apoptotic cells was significantly higher in the urinary bladder epithelium of MT-I/II null mice than in dose-matched wild-type mice exposed to DMA(V) (Jia et al. 2004).

## Genetic Damage and Apoptosis Induction by Arsenic Compounds

**Figure 2:**
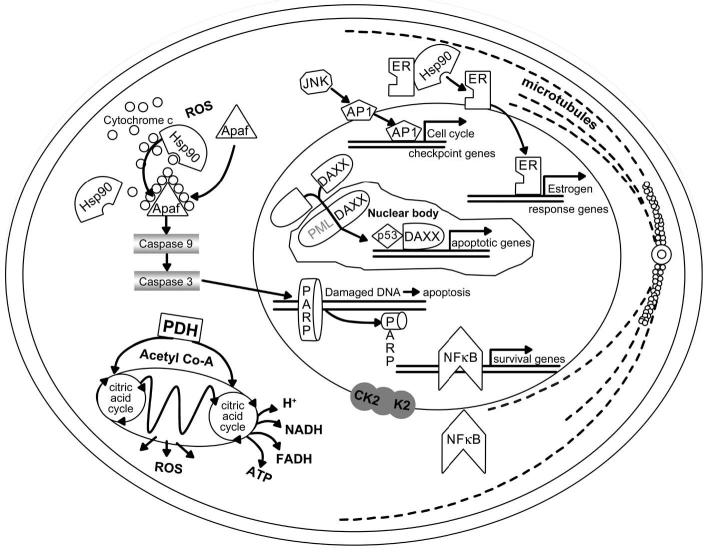
Cellular targets of arsenic trioxide action, with multiple pathways in malignant cells resulting in apoptosis or in the promotion of differentiation. Potential molecular targets for arsenic trioxide and arsenite are shown in gray. Abbreviations: AP1, activator protein-1; Apaf, apoptotic protease-activating factor; CK_2_, casein kinase; Co-A, coenzyme A; DAXX, death-associated protein; ER, estrogen receptor; FADH, flavin adenine dinucleotide; PARP, poly-(ADP-ribose)-polymerase; PML, promyelocytic leukemia. Modified from Miller et al. (2002) with permission from the American Association for Cancer Research.

Arsenite is widely used as a chemotherapeutic agent for the treatment of several human diseases. Arsenic trioxide has been used as a mitochondria-targeting drug in acute promyelocytic leukemia (Jimi et al. 2004; Lau et al. 2004; Miller et al. 2002; Rojewski et al. 2004; Zhang et al. 1999). Thus, arsenite and arsenic trioxide are cytotoxic (Jimi et al. 2004; Lau et al. 2004) and are capable of triggering apoptosis (Akao et al. 2000; Cai et al. 2003; Iwama et al. 2001; Shen et al. 2000; Zhang et al. 1999). Cellular targets of arsenic trioxide action are presented in Figure 2. Arsenic facilitates profound cellular alterations, including induction of apoptosis, inhibition of proliferation, stimulation of differentiation, and inhibition of angiogenesis via numerous pathways. The biologic effects of arsenic (principally the trivalent forms, arsenite and arsenic trioxide) may be mediated by reactions with closely spaced cysteine residues on critical cell proteins.

The cytotoxic potential of arsenic trioxide leads to decreased mitochondrial membrane potential, fragmented DNA, and finally to apoptotic cell death. Additionally, apoptosis induced by arsenic is mediated by a mechanism involving intracellular GSH-reactive oxidation (Akao et al. 2000; Jimi et al. 2004; Zhang et al. 1999).

At the molecular level of the cellular response, arsenite is able to up-regulate or down-regulate several proteins involved in different physiologic and pathologic pathways. In rat lung epithelial cells treated with arsenite, 7 of 1,000 proteins changed expression levels significantly. The up-regulated proteins were mostly heat-shock proteins (HSPs) and anti-oxidative stress proteins, including HSP70, aldose reductase, heme oxygenase-1, HSP27, ferritin light chain, and alphaB-crystallin. The glycolytic enzyme, glyceraldehyde-3-phosphate dehydrogenase, was down-regulated (Lau et al. 2004).

**Figure 3:**
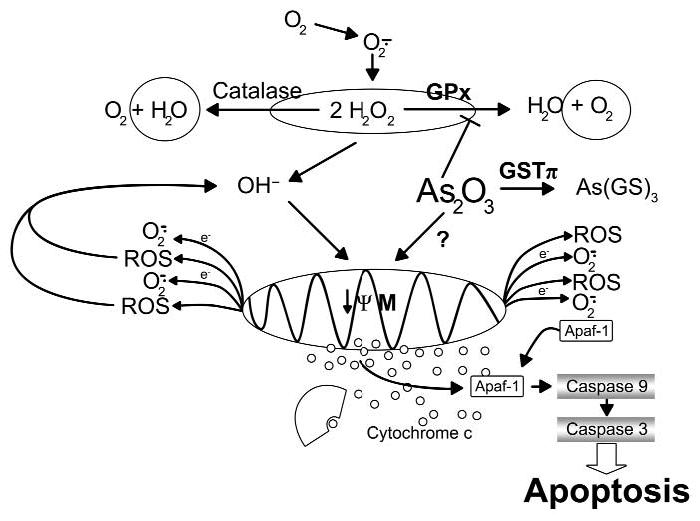
Apoptosis induced by arsenic trioxide by way of changes in mitochondrial membrane potential and increased H_2_O_2_ in cells; this lowers the mitochondrial membrane potential, leading to the release of cytochrome c and the activation of the caspase pathway. Abbreviations: ψM, mitochondrial inner transmembrane potential; Apaf-1, apoptotic protease-activating factor 1; GPx, glutathione peroxidase 1; GS, glutathione. Modified from Miller et al. (2002) with permission from the American Association for Cancer Research.

In addition, extracellular signal-regulated kinases ERK1 and ERK2 were completely inactivated, whereas p38 was found activated in human leukemia U937 cells treated with arsenic trioxide (As_2_O_3_). Experiments with transfected cells that expressed constitutively activated MAPK kinase MEK1 and a specific inhibitor of p38 have shown that inactivation of ERKs and activation of p38 might be associated with the induction of apoptosis by arsenic trioxide (Iwama et al. 2001). In contrast to the inactivation of ERKs and the activation of p38, activation of JNK by As_2_O_3_ appeared to protect cells against the induction of apoptosis. However, treatment of U937 cells with As_2_O_3_ also caused the Ca^2+^-dependent production of superoxide, intracellular acidification, and a decrease in the mitochondrial membrane potential at the early stages of apoptosis. These changes preceded the release of cytochrome c from mitochondria and the activation of caspase-3 (Figures 2 and 3) (Iwama et al. 2001; Miller et al. 2002).

Arsenic trioxide induces apoptosis in various cancer cells via complex mechanisms, which seem to be cell type dependent (Cai et al. 2003; Miller et al. 2002). Involvement of caspase 3 and caspase 8 was shown together with the down-regulation of Bcl-2 protein (Akao et al. 2000; Miller et al. 2002). A tight link between As_2_O_3_-induced apoptosis and mitotic arrest was recently shown by Cai et al. (2003), the latter being one of the common mechanism for As_2_O_3_-induced apoptosis in cancer cells. Arsenic can either enhance or reduce nitric oxide (NO) production, depending on the type of cell, the species, and dose of arsenical tested. The mechanisms of how arsenic increases or decreases NO production remain unclear (Gurr et al. 2003).

The Janus kinase (JAK)-signal transduction and activation of transcription (STAT) pathway is an essential cascade for mediating normal functions of different cytokines in the development of the hematopoietic and immune systems (Cheng et al. 2004). It has been suggested that arsenic-induced MAPK signal transduction leads to activation of transcription factors such as activator protein-1 (AP-1) and nuclear factor-κB (NFκB), which in turn alters gene expression (Yang and Frenkel 2002). This might be associated with the carcinogenicity of arsenic.

Ma et al. (1998) studied apoptosis in NB4 cells induced by sodium arsenite and arsenate using flow cytometry and DNA gel electrophoresis. The authors concluded that arsenite and arsenate induced apoptosis in NB4 cells by two different mechanisms: at low doses, arsenic might directly induce apoptosis through regulation of the cell cycle checkpoint, whereas at high doses it might directly induce apoptosis, but in this case Bcl-2 might not play an important role. Thus, the chemical valence of arsenic in a compound might be related to the efficiency of arsenical-induced apoptosis (Ma et al. 1998).

Woo et al. (2002) reported that HeLa cells underwent apoptosis in response to As_2_O_3_, accompanied by a decrease in mitochondrial membrane potential and caspase-3 activation. Overexpression of Bcl-2, however, prevented the dissipation of mitochondrial membrane potential, subsequently protecting the cells from As_2_O_3_-induced apoptosis. Arsenic trioxide increased the cellular content of ROSs, especially hydrogen peroxide, and the antioxidant *N*-acetyl-l-cysteine. Furthermore, incubation of the cells with catalase resulted in significant suppression of As_2_O_3_-induced apoptosis. The above results indicate that the induction of apoptosis in HeLa cells by arsenic trioxide include an early decrease in cellular mitochondrial membrane potential and an increase in ROS content, predominantly H_2_O_2_, followed by caspase-3 activation and DNA fragmentation (Miller et al. 2002; Woo et al. 2002).

**Figure 4:**
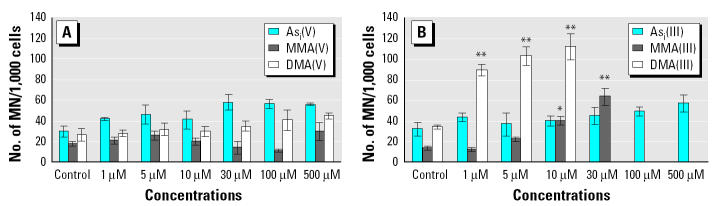
Micronucleus formation in CHO cells after treatment of cells with (*A*) As_i_(V), MMA(V), or DMA(V) and (*B*) As_i_(III), MMA(III), or DMA(III). The cells were incubated with the arsenic species for 1 hr. Two thousand binucleated cells were evaluated for micronucleus induction in each case. Data from Dopp et al. (2004b).
*p* ≤ 0.01, and
*p* ≤ 0.001, Student *t*-test.

For decades, arsenic has been considered a nongenotoxic carcinogen because it is only weakly active or, more often, completely inactive in bacterial and mammalian cell mutation assays. In recent studies, methylated metabolites of inorganic arsenic have been extensively investigated because of their high cytotoxic and genotoxic potential. Trivalent dimethylated arsenic, which can be produced by the metabolic reduction of DMA, has attracted considerable attention from the standpoint of arsenic carcinogenesis. Several groups have shown that DMA(III) is highly genotoxic compared with the pentavalent species and inorganic arsenic (e.g., Dopp et al. 2004b; Schwerdtle et al. 2003) (Figure 4).

Ochi et al. (1996) studied the induction of apoptosis caused by the methylated arsenic species. These authors showed that DMA(V) induces apoptosis in cultured human HL-60 cells at concentrations of 1–5 mM after an incubation period of 18 hr. On the other hand, Cohen et al. (2002) showed that *in vivo* administration of DMA(V) results in cytotoxicity with necrosis, followed by regenerative hyperplasia of the bladder epithelium. DMA(V) exerted differential antiproliferative and cytotoxic activity against leukemia and multiple myeloma cells, with no significant effect on normal progenitor cells (Duzkale et al. 2003).

In comparison with the trivalent inorganic arsenic form, therapeutic concentrations of As_2_O_3_ (1–2 μM) had dual effects on malignant lymphocytes: *a*) inhibition of growth through adenosine triphosphate (ATP) depletion and prolongation of cell cycle time, and *b*) induction of apoptosis (Zhu et al. 1999).

Zhang et al. (1999) suggested that the increase in intracellular Ca^2+^ is related to the sensitivity of human cells to As_2_O_3_ exposure, indicating that a critical intracellular Ca^2+^ signal transduction pathway could be involved in As_2_O_3_-mediated cell death.

## The Toxicity of Arsenicals Is Related to Calcium Homeostasis Disturbances

In order to explore the early apoptotic signal messengers and the apoptotic pathway, the morphologic and functional changes of mitochondria were examined in a study by Shen et al. (2002b). The content of NO and free calcium ions (Ca^2+^) was measured over the course of apoptosis induction after exposure with As_2_O_3_ in esophageal carcinoma cells (SHEEC1). SHEEC1 cells were exposed to As_2_O_3_ (1, 3, and 5 μmol/L), and after 0, 2, 4, 8, 12, and 24 hr, the fluorescence intensity (FI) of rhodamine 123 (Rho123)-labeled cells was detected using a confocal laser scanning microscope for evaluation of the mitochondrial membrane potential. After adding arsenic trioxide, SHEEC1 cells showed characteristic morphologic and functional changes of mitochondria such as hyperplasia, disruption, and an accompanying decrease in transmembrane potential (FI of Rho123 decreased). The Ca^2+^ level increased immediately after adding As_2_O_3_, and the NO concentration increased in a step-wise manner up to 24 hr. At this time the cells appeared to have an apoptotic morphology. The results of Shen et al. (2002a, 2002b) suggest that by inducement of As_2_O_3_-increased Ca^2+^ and NO levels, the apoptotic signal messengers initiate the mitochondria-dependent apoptotic pathway.

**Figure 5:**
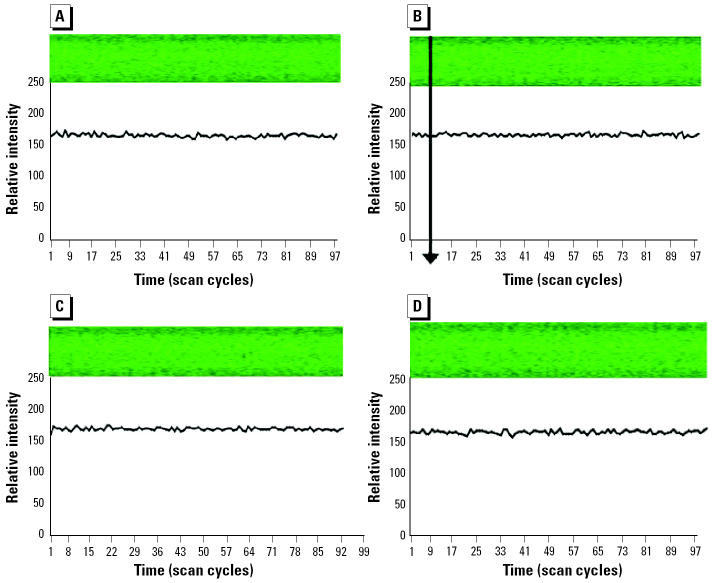
Intracellular calcium changes (relative intensity units measured by confocal laser scanning microscopy) in HeLa S3 cells after application of HEPES buffer (negative control). (*A*) Control. (*B*) Control and application of HEPES buffer (indicated by arrow). (*C*) Control after 1 min. (*D*) Control after 3 min. The incubation buffer did not modify the initial level of fluorescence intensity. No photo bleaching occurred. Data from Florea (2004).

**Figure 6:**
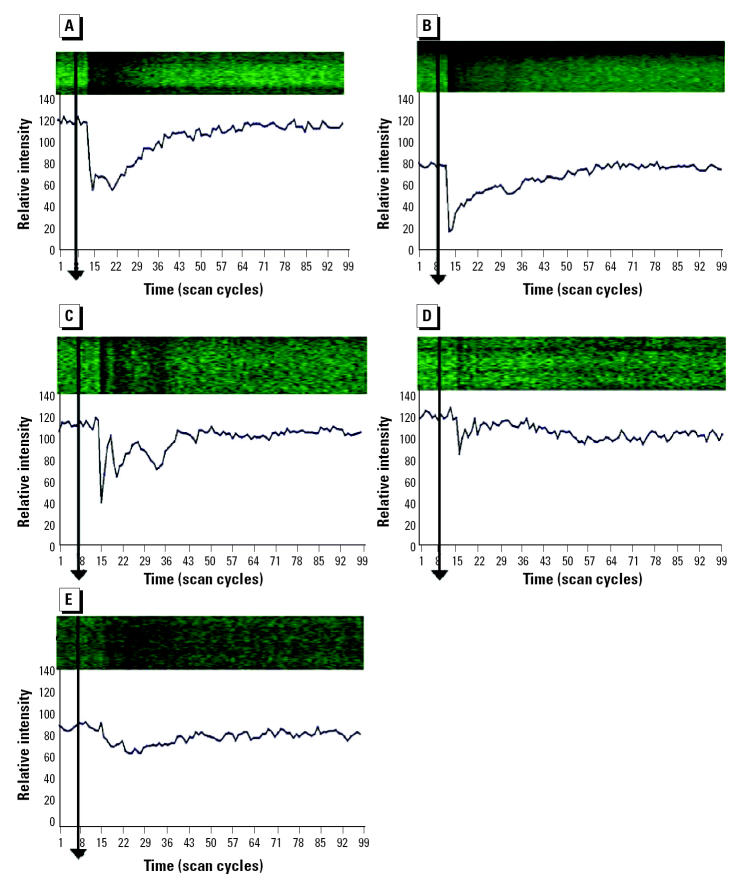
Intracellular calcium changes (relative intensity units measured by confocal laser scanning microscopy) in HeLa S3 cells after application of 0.5 mM of different arsenic species (indicated by arrows). (*A*) As_i_(III); (*B*) As_i_(V); (*C*) MMA(V); (*D*) DMA(V); (*E*) TMAO. Note the drop in the fluorescence signal immediately after application (Florea 2004).

In previous experiments (Florea 2004) we assessed inorganic As_i_(III) and As_i_(V), as well as MMA(V), DMA(V), and TMAO(V) (0.5 mM concentration) for early disturbances in calcium homeostasis in HeLa S3 cells within the first few seconds after application. If calcium homeostasis was disturbed, a drop in the fluorescence signal of the dye was recorded by confocal laser scanning microscopy. The drop was transient, and the signal returned rapidly to the initial level within 20 sec (Figures 5 and 6). These calcium signals might occur as active efflux from the cell to the exterior (energy consuming) or as deregulation of other ion transports. A mechanism via membrane receptor activation or membrane damage cannot be excluded (Florea 2004).

Recently, the original calcium hypothesis has been modified, taking into account that cell death is induced under experimental conditions not only by a rise in cytoplasmatic calcium but also when cytoplasmatic calcium activity drops below physiologic levels (Paschen 2003). Cellular stimulation can lead to activation of different signal transduction mechanisms, such as alterations of the cytoplasmatic levels of different ions. Cell alkalization slightly decreases the intracellular Ca^2+^ concentration due to an efflux of Ca^2+^ from the cell. Elevation of pH, however, increases Ca^2+^ either in the presence or absence of external Ca^2+^ (Cabado et al. 2000). In contrast to these findings, Kauppinen et al. (1989) reported a study involving cortical synaptosomes in the guinea pig. Cytosolic calcium drops were seen in this study in the absence of Ca^2+^ in the external solution and were related to an increased glucose utilization (Kauppinen et al. 1989). On the other hand, Buja et al. (1993) suggested that the initial modifications of cellular metabolism and calcium homeostasis may activate major pathways leading to a loss of membrane integrity by *a*) membrane phospholipid degradation, *b*) production of amphipathic lipids, *c*) damage of the cyto-skeleton, and *d*) generation of toxic oxygen species and free radicals.

## Cellular Mechanisms of Intracellular Calcium Changes in Relation to Genetic Damage

Regulation of intercellular and intracellular signaling is fundamental for survival and death in biologic organisms; the systems that control ion movements across cell membranes are essential for cell survival. A deregulation of channels or pumps can cause events that lead to cell death. Apoptosis can be caused by loss of Ca^2+^ homeostatic control but can also be positively or negatively controlled by changes in Ca^2+^ distribution within intracellular compartments. It was shown that even non-disruptive changes in Ca^2+^ signaling could have adverse effects, including alterations in cell proliferation and differentiation, as well as in the modulation of apoptosis (Orrenius et al. 2003).

Cellular Ca^2+^ import through the plasma membrane occurs largely by receptor-operated and voltage-sensitive channels. Once inside the cell, Ca^2+^ can either interact with Ca^2+^-binding proteins or become sequestered to the endoplasmic reticulum (ER) or mitochondria, reaching millimolar levels. Ca^2+^ levels in the ER are regulated by Ca^2+^-ATPase pumps, inositol 1,4,5-trisphosphate (IP3) receptors, ryanodine receptors, and Ca^2+^-binding proteins (Orrenius et al. 2003). Thus, the mitochondrial permeability transition is involved in apoptotic cell death, in that it releases pro-apoptotic proteins from the mitochondria into the cytosol where, with the aid of cellular ATP, they complete the apoptotic cascade. The complexity of the regulation of Ca^2+^ inside the cell is probably because mitochondria are able to modulate the amplitude and shape of Ca^2+^ signals (Babcock et al. 1997). However, mitochondria contribute to both apoptotic and necrotic cell death (Nieminen 2003).

It was previously demonstrated by Lui et al. (2003) that tubules, in a vertical or horizontal orientation, extend deep inside the nucleus of HeLa cells. These extensions, together with the nuclear envelope and ER, physically form a spatial network. For Ca^2+^ signaling, the nuclear tubules provide a fast transport system to direct the release of IP3 and Ca^2+^ from the cytosol to the nucleus or vice versa. The lumen of the nuclear tubules contains many organelles, including mitochondria that move in and out of the nuclear tubules. To reduce Ca^2+^ overloading, mitochondria can take up a considerable amount of Ca^2+^ inside the nuclear tubules (Lui et al. 2003). Li et al. (1998) described that oscillations in cytosolic calcium at physiologic rates maximize gene expression depending on IP3. Spikes of cytosolic calcium were able to stimulate gene expression via the nuclear factor of activated T cells (Li et al. 1998).

Another study by Liu and Huang (1996) demonstrated that calcium ions are accumulated in the nuclei of Chinese hamster ovary (CHO)-K1 cells after arsenite treatment. These observed effects were related to disturbances in intracellular calcium homeostasis and with arsenite-induced cytotoxicity and micronucleus formation. A modulation of the calcium level within the nucleus might have toxic effects leading to DNA damage and/or inhibition of DNA repair function. Some authors have shown that micronucleus formation (expressing DNA damage) as well as induction of mitotic disturbances is strongly correlated with disturbances of calcium homeostasis (Dopp et al. 1999; Liu and Huang 1996; Xu et al. 2003). The correlation between DNA damage and calcium homeostasis disturbances was supported for As_i_(III) when Liu and Huang (1997) observed an elevation of intracellular calcium after arsenite treatment. These authors showed that calcium ions play an essential role in arsenite-induced genotoxicity and concluded that arsenite exposure perturbs intracellular calcium homeostasis and activates protein kinase C activity in a dose-dependent manner.

Bucki and Gorski (2001) even suggested that the nucleoplasmic calcium concentrations ([Ca^2+^]_n_) may be regulated independently of that of cytosolic Ca^2+^. IP3 and cyclic ADP-ribose are the major factors responsible for Ca^2+^ release into the nucleus from the perinuclear space. [Ca^2+^]_n_ is involved in the regulation of many events in the nucleus, such as gene expression, DNA replication, DNA repair, chromatin fragmentation in apoptosis, and modulation of an intranuclear contractile system. The importance of a precise cellular Ca^2+^-level regulation for an optimal DNA repair process was mentioned already by Gafter et al. (1997). Bugreev and Mazin (2004) showed that the human Rad51 protein, which plays a key role in homologous recombination and DNA repair, is dependent upon the intra-cellular calcium level. Arsenic and its methylated derivatives are able to modulate DNA repair processes (e.g., Andrew et al. 2003; Hartwig 1998; Hartwig et al. 2003) and gene expression (e.g., Elbekai and El-Kadi 2004; Wu et al. 2003). A possible correlation between inhibition of DNA repair function as well as changed gene expression profiles caused by arsenicals and disturbed intracellular calcium homeostasis requires further investigations.

## Conclusions

Epidemiologic evidence suggests that exposure to inorganic arsenic causes cancer (e.g., National Research Council 1999). However, the mechanism of arsenic carcinogenesis is still unclear. A complicating factor receiving increasing attention is that arsenic is bio-methylated to form various metabolites. Methylated arsenic species are able to induce genomic damage as well as apoptosis *in vivo* and *in vitro*. Most research has been done with DMA(V) because it has neurotoxic effects and induces bladder cancer in rats and apoptosis in cultured human cells (Jia et al. 2004; Namgung and Zia 2001; Xie et al. 2004). The conjugation of DMA(V) with cellular GSH appears to be of mechanistic significance. More research is needed to determine the role of intracellular GSH and methylation in the toxicity of arsenicals in chronic arsenic poisoning or in cases where arsenicals are used as chemotherapeutics.

Several investigations have shown that DMA(V) exposure causes oxidative stress, DNA damage, and specific induction of apoptosis in target organs of arsenic carcinogenesis (Jia et al. 2004; Sakurai et al. 2004), which may be attributable to the mechanism(s) of arsenic-induced carcinogenesis in rodents. Compared with the pentavalent methylated arsenic species, the trivalent species are even more reactive and cause calcium homeostasis disturbances, oxidative stress, DNA damage, and apoptosis to a higher extent. The involvement of internal calcium stores, particularly mitochondria, can be assumed. This specific area requires further research. Also, more research should focus on the cellular effects of arsenic metabolites, which are generated inside the cell and may cause cellular damage at much lower concentrations than the inorganic arsenic species.

Many studies in the literature describe the effects of arsenite and arsenic trioxide on cellular targets, because these chemicals are or have been used as chemotherapeutic agents for the treatment of several human diseases. Apoptosis induction caused by As_2_O_3_ has been shown to be related with changes of the intracellular calcium concentration (e.g., Akao et al. 2000; Cai et al. 2003). The intracellular Ca^2+^ level increases immediately after adding As_2_O_3_. The initiation of the mitochondria-dependent apoptotic pathway was suggested (Iwama et al. 2001; Miller et al. 2002).

A precise cellular Ca^2+^-level regulation is also necessary for optimal DNA repair processes, DNA replication, and gene expression. Arsenicals are able to modulate these processes. A direct correlation between genotoxic effects caused by arsenicals and disturbances of intracellular calcium concentration is partially proven but requires further investigations.
